# Draft Genome Sequence of *Lactobacillus rhamnosus* 2166

**DOI:** 10.1128/genomeA.01222-13

**Published:** 2014-02-20

**Authors:** Andrey V. Karlyshev, Vyacheslav G. Melnikov, Igor V. Kosarev, Vyacheslav M. Abramov

**Affiliations:** a School of Life Sciences, Faculty of Science, Engineering and Computing, Kingston University, Kingston upon Thames, United Kingdom; b International Science and Technology Center, Moscow, Russia; c Institute of Immunological Engineering, Lyubuchany, Moscow Region, Russia

## Abstract

In this report, we present a draft sequence of the genome of *Lactobacillus rhamnosus* strain 2166, a potential novel probiotic. Genome annotation and read mapping onto a reference genome of *L. rhamnosus* strain GG allowed for the identification of the differences and similarities in the genomic contents and gene arrangements of these strains.

## GENOME ANNOUNCEMENT

Similar to other lactic acid-producing bacteria, *Lactobacillus rhamnosus* was found to be a promising probiotic with beneficial properties for human health (1, 2). According to the Web of Science database, the number of studies mentioning this bacterium doubled in a recent 5-year period (2007 to 2012). One of the most extensively studied strains is *L. rhamnosus* GG, which is widely used in the dairy industry in some countries. An analysis of several commercial isolates of *L. rhamnosus* revealed that they are almost identical to strain GG (3). Rare cases of *L. rhamnosus*-associated bacteremia were not linked with increased consumption of products containing this bacterium (4, 5).

A search for strains with improved properties and reduced side effects might be assisted by comparative genomics studies. A recent comparative analysis of the genomes of 100 strains of this species by mapping onto the genome of *L. rhamnosus* GG revealed significant interstrain variation in genomic content and organization (6). However, most of these strains were only partially characterized in terms of their biochemical or probiotic properties, making it difficult to link genetic and phenotypic features.

Here, we describe a draft genome sequence of *L. rhamnosus* 2166, which showed superior characteristics in a comparison with other strains of this species (patent pending). The strain was isolated from a dairy product based on soured goat milk and identified at the Institute of Engineering Immunology, Moscow Region, Russia, in 2011. One remarkable feature of this strain is a highly efficient ability to ferment vegetable juices, particularly beetroot juice. Animal experiments demonstrated beneficial effects of these fermentation products on cecal microbial activity (7).

Sequencing reads, generated by Ion Torrent PGM with Chip 314, were assembled using the CLC Genomics Workbench and Ion Torrent programs. The contigs were joined by mapping onto the genome of *L. rhamnosus* strain GG (ATCC 53103) using the MG Finishing Module of CLC Genomics Workbench to generate 84 contigs (1,014 to 395,531 nucleotides [nt]). The total genome assembly (3,016,360 nt with 17.08× coverage) and G+C content (46.6%) were in good agreement with the respective figures for the published genome sequences of other strains of this species (2.52 to 3.16 Mb and 46.3 to 46.9%).

The genome sequence was annotated using NCBI GenBank and RAST (8) software. One remarkable feature found was the presence of a large number of sugar metabolism-related genes. Among the other genes detected were those involved in fibronectin-binding protein and colicin V production, as well as the genes responsible for resistance to β-lactam antibiotics.

Mapping of reads onto the genome of the test strain GG revealed the presence of the pilin genes *spaD, spaE*, and *spaF*, while some pilin genes (including the *spaC* gene, encoding a mucus-binding protein) (9) were missing. A number of tandemly arranged genes involved in capsular polysaccharide biosynthesis in strain GG were also missing in strain 2166. Among the other genes missing in the test strain were those encoding Lon protease, various phage-related proteins, and IS*5* and IS*150*/IS*3*-related transposases.

The features identified in the genome of *L. rhamnosus* 2166 will assist in a better understanding of its beneficial properties.

### Nucleotide sequence accession numbers.

This whole-genome shotgun project has been deposited at DDBJ/EMBL/GenBank under the accession no. AVFG00000000. The version described in this paper is version AVFG02000000.
